# Global analysis of prokaryotic tRNA-derived cyclodipeptide biosynthesis

**DOI:** 10.1186/s12864-018-4435-1

**Published:** 2018-01-15

**Authors:** Michael A. Skinnider, Chad W. Johnston, Nishanth J. Merwin, Chris A. Dejong, Nathan A. Magarvey

**Affiliations:** 1 Department of Biochemistry and Biomedical Sciences, Michael G. DeGroote Institute for Infectious Disease Research, McMaster University, Hamilton, ON L8S 4K1 Canada; 2 Department of Chemistry and Chemical Biology, Michael G. DeGroote Institute for Infectious Disease Research, McMaster University, Hamilton, ON L8S 4K1 Canada

**Keywords:** Cyclodipeptide synthase, Genome mining, Secondary metabolism

## Abstract

**Background:**

Among naturally occurring small molecules, tRNA-derived cyclodipeptides are a class that have attracted attention for their diverse and desirable biological activities. However, no tools are available to link cyclodipeptide synthases identified within prokaryotic genome sequences to their chemical products. Consequently, it is unclear how many genetically encoded cyclodipeptides represent novel products, and which producing organisms should be targeted for discovery.

**Results:**

We developed a pipeline for identification and classification of cyclodipeptide biosynthetic gene clusters and prediction of aminoacyl-tRNA substrates and complete chemical structures. We leveraged this tool to conduct a global analysis of tRNA-derived cyclodipeptide biosynthesis in 93,107 prokaryotic genomes, and compared predicted cyclodipeptides to known cyclodipeptide synthase products and all known chemically characterized cyclodipeptides. By integrating predicted chemical structures and gene cluster architectures, we created a unified map of known and unknown genetically encoded cyclodipeptides.

**Conclusions:**

Our analysis suggests that sizeable regions of the chemical space encoded within sequenced prokaryotic genomes remain unexplored. Our map of the landscape of genetically encoded cyclodipeptides provides candidates for targeted discovery of novel compounds. The integration of our pipeline into a user-friendly web application provides a resource for further discovery of cyclodipeptides in newly sequenced prokaryotic genomes.

**Electronic supplementary material:**

The online version of this article (10.1186/s12864-018-4435-1) contains supplementary material, which is available to authorized users.

## Background

Small molecule natural products have historically been a primary source of industrial and pharmaceutically important agents [1]. The privileged scaffolds of these compounds have been optimized by evolution for targeted interactions with biological macromolecules, in order to provide a fitness advantage for their producers [2]. As a result, they represent the basis for the majority small molecule drugs currently in clinical use [1]. A class of natural products that have attracted considerable interest in recent years for their bioactivities are the cyclodipeptides [3]. The scaffolds of these compounds can be accessed either by nonribosomal peptide synthases (NRPSs), or alternatively via cyclodipeptide synthases (CDPSs) [4]. Both classes of enzymes are typically located within a cluster of genes involved in the biosynthesis and tailoring of the cyclodipeptide product [5]. Although NRPSs have been extensively studied, relatively less is known about the CDPSs. These small enzymes catalyze cyclodipeptide biosynthesis from two aminoacyl-tRNA substrates [6]. In several well-studied biosynthetic pathways, the resulting cyclodipeptides are further modified by associated tailoring enzymes to yield the final product [5]. The few cyclodipeptides known to be biosynthesized via CDPS-dependent routes are noted for their antibacterial activities (e.g., albonoursin [7] and nocazine [8]), while pulcherriminic acid is an iron chelating agent, and mycocyclosin may be essential for Mycobacterium tuberculosis activity [4]. More generally, bioactive cyclodipeptides include the antibiotic bicyclomicin, used as a food additive to prevent diarrhea in livestock [9], the cytotoxic agent neihumicin [10], and the immunosuppressive agent gliotoxin [11], as well as other small molecules with antifungal, antiviral, and anti-inflammatory activities [12].

With the advent of next-generation sequencing, and the attendant growth of microbial genome sequence data in the public domain, simple BLAST-based searches have revealed large numbers of cryptic CDPS clusters in prokaryotic genomes [13]. However, whereas a large number of tools that have been developed to identify NRPS biosynthetic gene clusters and to predict the chemical structures of the genetically encoded peptides, at present CDPSs identified within genome sequence data cannot be linked to their cyclodipeptide products except via manual annotation by experts. As a result, no systematic effort to describe the chemical space encoded within sequenced microbial genomes has been undertaken. It remains unclear what proportion of genetically encoded CDPSs produce novel cyclodipeptides, or how experimental resources should be prioritized to facilitate discovery of bioactive products. The development of user-friendly tools to uncover connections between sequence data and cyclodipeptide products could help guide the identification of novel, bioactive cyclodipeptides.

Here, we describe an algorithm for the discovery and classification of CDPS biosynthetic gene clusters, identification of CDPS active site residues, and prediction of both aminoacyl-tRNA substrates and final, tailored cyclodipeptide products. We validate our pipeline and integrate it into a user-friendly web application accessible to non-specialists. We then use this tool to conduct a global analysis of tRNA-derived cyclodipeptide biosynthesis within 93,107 prokaryotic genomes. This analysis defines the chemical space occupied by genetically encoded cyclodipeptides, and shows that a considerable area of this chemical space remains undiscovered. Our algorithm and survey of cyclodipeptide biosynthesis provide resources for targeted discovery of new cyclodipeptides.

## Results and discussion

### Genomic prediction of cyclodipeptide synthase products

**Fig. 1:**
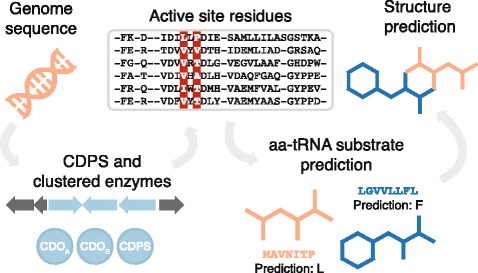
Schematic overview of an algorithm for tRNA-derived cyclodipeptide identification and prediction. Given a microbial genome sequence as input, our algorithm uses a hidden Markov model to identify CDPSs and cluster them with surrounding biosynthetic and resistance enzymes, including tailoring enzymes specific to cyclodipeptide biosynthesis. CDPS active site residues are identified by multiple sequence alignment to a large database of CDPS sequences, and their aminoacyl-tRNA substrates are predicted by a naive Bayes classifier. Finally, tailoring reactions are executed to generate a predicted cyclodipeptide chemical structure

We developed an algorithm to identify CDPSs, classify their subfamilies, identify active sites and predict their aminoacyl-tRNA substrates, and predict the final chemical structures of their corresponding products (Fig. 1, Methods). CDPSs are identified using a hidden Markov model developed for this study, and their sequences are subsequently analyzed using a set of subfamily-specific hidden Markov models to classify CDPSs as members of the NYH, XYP, or SYQ subfamilies [14]. These phylogenetically distinct subfamilies were characterized by a recent study [14], and are distinguished on the basis of the conserved residues within the active site of each subfamily of CDPSs. Active site residues are identified by multiple sequence alignment with a large library of CDPSs, and their aminoacyl-tRNA substrates are predicted using a naive Bayes classifier. Each CDPS is clustered with neighboring biosynthetic enzymes, and the chemical structure of the CDPS product is predicted by cyclization of the CDPS substrates and execution of any tailoring reactions. The algorithm is integrated within the PRISM web application [15], and is publicly available at https://magarveylab.ca/prism. To supplement existing generic tailoring reactions within PRISM (e.g., N-methylation or halogenation), we constructed new hidden Markov models for all known CDPS tailoring enzymes, in order to maximize the accuracy of chemical structure predictions (Additional file 1**:** Table S1).

### Validation of tRNA-derived cyclodipeptide identification, classification and prediction

Having developed a pipeline for automated identification and structure prediction of genetically encoded tRNA-derived cyclodipeptides, we next sought to validate the performance of each component of the algorithm. First, we assessed the ability of our CDPS hidden Markov model to identify new CDPS clusters by leave-one-out cross-validation, systematically excluding every sequence used to build the model in turn and testing the ability of the model to identify the withheld sequence. All 273 withheld sequences were identified above the bitscore cutoff, suggesting this model is capable of sensitively identifying new CDPS clusters.

Second, we evaluated the ability of our library of subfamily-specific CDPS subtype hidden Markov models to predict the phylogenetic subfamily of withheld sequences. Withheld sequences of NYH- and XYP-family CDPSs were classified with 100% accuracy; since the SYQ model was derived from a single sequence, the performance was 0% with this sequence withheld.

**Fig. 2:**
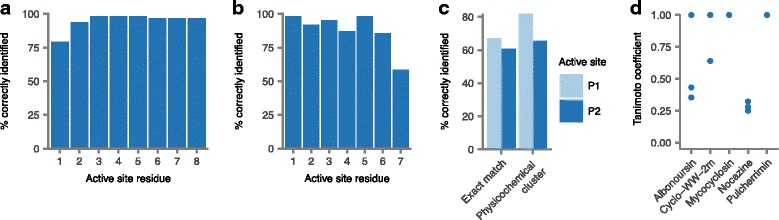
Validation of cyclodipeptide synthase chemical structure prediction in PRISM. **a–b** Accuracy of CDPS active site residue prediction in LOOCV for the P1 (c) and P2 (d) active sites. **c** Accuracy of CDPS aminoacyl-tRNA substrate prediction at the P1 and P2 active sites in LOOCV. **d** Tanimoto coefficient accuracy of chemical structure prediction for five known cluster-compound pairs

Third, we evaluated the accuracy of active site residue prediction using multiple sequence alignment (Fig. 2c–d). In total, 91.6% of active site residues within withheld sequences were correctly predicted, indicating that multiple sequence alignment to a large CDPS sequence database is an effective strategy for active site residue identification (Additional file 2**:** Table S2). Active site residues that were incorrectly identified were predominantly located near the start or end of the CDPS sequence, where higher levels of sequence divergence occasionally resulted in predicted active site residues that were misaligned by one or more amino acids relative to those manually identified by Jacques et al. [14], on the basis of CDPS secondary structures. Although manual adjustment is expected to produce more accurate active site residue identification, it is impractical within an automated workflow and on a scale suitable for the analysis of over 90,000 prokaryotic genomes presented here; we therefore considered the accuracy acceptable for downstream analysis. However, the possibility that the manual adjustments of Jacques et al. do not actually represent the true active site residues cannot be excluded in the absence of experimental structural evidence.

Fourth, we assessed the ability of our naive Bayes classifier to correctly predict the aminoacyl-tRNA substrates of each withheld CDPS active site (Fig. 2e). Amino acids activated by active sites P1 and P2, were predicted with accuracies of 67.2% and 60.7%, respectively, corresponding to 8.1- and 7.3-fold enrichment over random expectation. When considering amino acids within the same physicochemical clusters defined by Rausch et al. [16] as matches, the predictive accuracy rose to 74.1% for P1 and 66.7% for P2. The overall accuracy was therefore 70.4%. These results suggest that the naive Bayes classifier is able to predict the aminoacyl-tRNA substrates of each active site with good accuracy. We anticipate that the continued addition of active site/aminoacyl-tRNA pairs to the training dataset with the discovery of new cyclodipeptides should further improve the accuracy.

Finally, we evaluated the accuracy of cyclodipeptide chemical structure predictions for five cyclodipeptides with known clusters using the Tanimoto coefficient (Fig. 2f). PRISM correctly predicted the structure of the corresponding product with 100% accuracy for four of five clusters; for the fifth cluster, nocazine, the structure of a minor product (nocazine E) was predicted. The average median Tanimoto coefficient across all five clusters was 0.71, similar to that reported for ribosomally synthesized and posttranslationally modified peptides (RiPPs) [17], and higher than that for nonribosomal peptides, polyketides, bisindoles, aminocoumarins, or phosphonates [18]. Taken together, these results indicate that our pipeline is capable of identifying and predicting the structures of genetically encoded cyclodipeptides with a high degree of accuracy.

### Genomic analysis of tRNA-derived cyclodipeptide biosynthesis

Having developed and validated an algorithm for tRNA-derived cyclodipeptide cluster identification and structure prediction, we leveraged this algorithm to conduct a global analysis of tRNA-derived cyclodipeptide biosynthesis in prokaryotes. We used PRISM to analyze 93,107 prokaryotic genomes downloaded from National Center for Biotechnology Information (NCBI) Genome (March 2017), and identified 6580 cyclodipeptide clusters. The vast majority (98.1%) of tRNA-derived cyclopeptide producer genomes contained only a single CDPS cluster. However, a small number of organisms were more prolific cyclodipeptide producers, with 27 encoding three CDPS clusters, and three organisms encoding four (Additional file 3**:** Figure S1a).

**Fig. 3:**
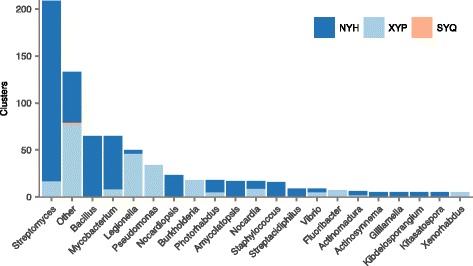
Unique tRNA-derived cyclodipeptide clusters in a sample of 93,107 prokaryotic genomes, organized by genus of producing organism. Genera with fewer than five cyclodipeptide clusters are marked as “Other”

To control for repetitive sequencing of cyclodipeptide producers, we used cd-hit [19] to filter CDPS sequences with 100% identity. Filtering redundant sequences revealed a set of 721 unique cyclodipeptide clusters, corresponding to 739 unique CDPSs (Additional file 4**:** Table S3), which were retained for further analysis. Among the 18 clusters with two CDPSs, 89% of CDPS pairs were within 2.5 kilobases (kb) (Additional file 3**:** Figure S1b–c), suggesting that the majority represent true clusters containing more than one CDPS, rather than spurious clusters composed of distant CDPSs. The distribution of CDPS clusters across bacterial genera revealed the abundance of these biosynthetic systems in organisms well known for their secondary metabolic capacity, in particular the Actinobacteria (Fig. 3). However, CDPS clusters were also identified in a number of Gram-negative bacteria, including *Burkholderia*, *Legionella*, and *Photorhabdus*.

### Chemical space of genetically encoded cyclodipeptides

**Fig. 4:**
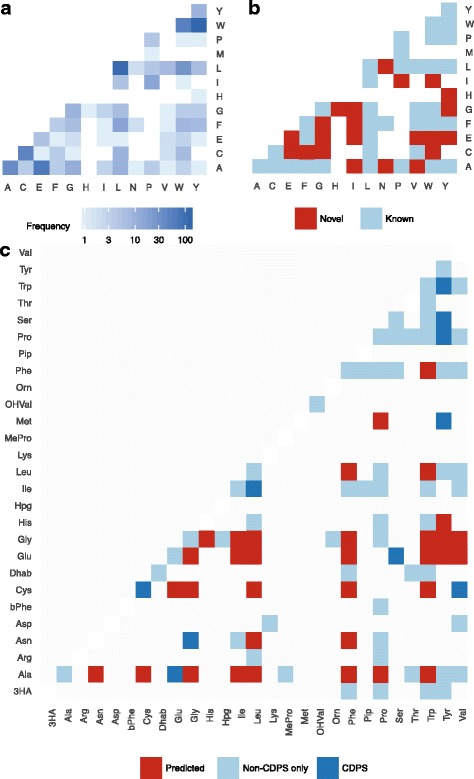
**a** Frequency of predicted tRNA-derived cyclodipeptides in a global analysis of prokaryotic CDPS biosynthesis. **b** Comparison of known and predicted novel genetically encoded tRNA-derived cyclodipeptides. **c** Chemocentric view of known NRPS- and CDPS-derived cyclodipeptides and predicted tRNA-derived cyclodipeptides. “CDPS” refers to cyclodipeptides known to be produced by characterized CDPS enzymes, while “Non-CDPS only” refers to naturally occurring cyclodipeptides that are not known to be produced by any characterized CDPS

We next sought to predict the chemical space occupied by genetically encoded cyclodipeptides. Analysis of predicted substrates at the P1 and P2 active sites revealed 59 of 99 total possible products within the set of 739 CDPSs (60%**,** Fig. 4a). The most common predicted aminoacyl-tRNA combinations were WY (141 CDPSs), LL (120 CDPSs), AE (72 CDPSs), WW (58 CDPSs), and CC (50 CDPSs) (Fig. 4a). In contrast, 18 aminoacyl-tRNA combinations were predicted only once. To evaluate the chemical diversity of genetically encoded cyclodipeptides in sequenced prokaryotes, we compared the predicted aminoacyl-tRNA combinations to the set of known CDPS products compiled by Jacques et al. [14] (Fig. 4b). In total, 27 predicted cyclodipeptides, or 37% of unique aminoacyl-tRNA combinations, represented novel products. Although further biochemical characterization is required to conclusively define the product(s) of each CDPS, this global view of cyclodipeptide biosynthesis suggests that even before considering biosynthetic tailoring reactions, a considerable fraction of genetically encoded cyclodipeptide chemical space remains unknown.

To gain a chemocentric view of cyclodipeptide biosynthesis, we supplemented the set of known tRNA-dependent cyclodipeptide products by identifying cyclic dipeptides within a set of 46,165 compounds, using GRAPE [20] to identify cyclic dipeptides by in silico retrobiosynthesis (Fig. 4c). This analysis revealed that many possible dipeptides are known to be produced only via CDPS-independent mechanisms. However, even within the broader landscape of all known cyclic dipeptides, a large fraction of CDPSs identified in existing genomic data are predicted to encode novel dipeptide products.

### Recurrent biosynthetic and non-biosynthetic domains in tRNA-derived cyclodipeptide biosynthesis

**Fig. 5:**
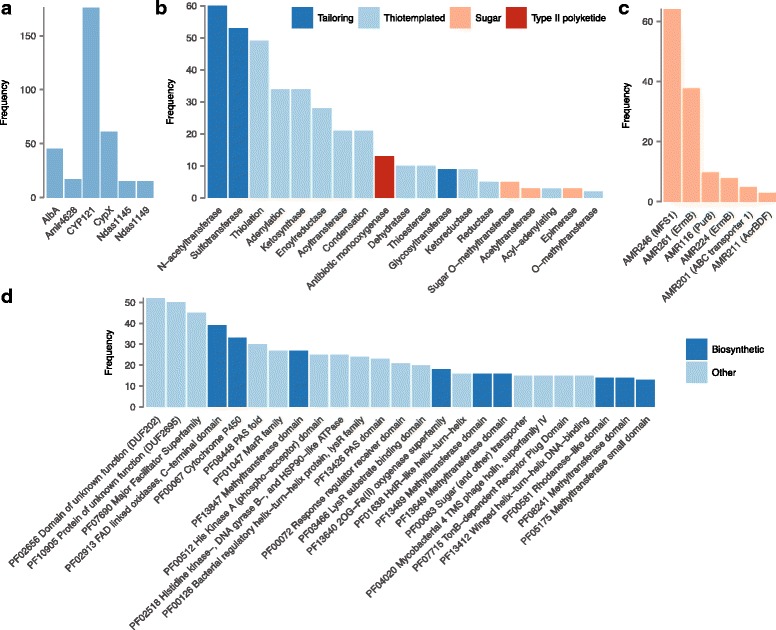
**a** Frequency of known cyclodipeptide tailoring reaction domains found in association with cyclodipeptide clusters. In all panels, frequency corresponds to the number of unique CDPS clusters in which the tailoring reaction was observed. **b** Frequency of other PRISM tailoring reaction domains found in association with cyclodipeptide clusters. Only domains found at least three times are shown. **c** Frequency of PRISM resistance domains found in association with cyclodipeptide clusters. Only domains found at least three times are shown. **d** Frequency of biosynthetic and non-biosynthetic Pfam domians found in association with cyclodipeptide clusters

To provide further insight into the chemical space occupied by genetically encoded cyclodipeptides, we catalogued the frequency of the five characterized cyclodipeptide tailoring reactions executed during structure prediction by PRISM (Fig. 5a). Notably, homologs of the mycocyclosin aryl carbon–carbon bond-forming oxidase CYP121 were found to be widely distributed in Mycobacteria and related Actinobacteria, nearly always in association with aromatic amino acids as predicted substrates. The presence of a CYP121 homolog in 176 of 721 cyclodipeptide clusters, a total of 24%, suggests that carbon–carbon bond formation between aromatic amino acids is a widespread tailoring reaction in cyclodipeptide biosynthesis. The pulcherriminic acid N-oxygenase was observed in a large number of *Bacillus* sequences, but was also found in related *Staphylococcus* Firmicutes, *Corynebacterium*, and *Photorhabdus luminescens*. Heterologous expression of the *P. luminescens* CDPS leads to production of the leucine cyclodipeptide precursor of pulcherriminic acid [3], providing further evidence that this biosynthetic pathway is more broadly distributed than had been previously suspected. Hits for the AlbA nitroreductase and Ndas1145 amide O- and N-methyltransferase were confined to Actinobacteria, while the Ndas1149 O-methyltransferase from the nocazine pathway was exclusively identified in *Nocardiopsis* spp. Among 42 AlbA hits, 23 were co-localized with CYP121, Ndas1145, or Ndas1149, suggesting that nearly half of the associated clusters encode albonoursin or related products, whereas the remaining half may be related to piperafazines, nocazines, neihumicin, or other methylated cyclodipeptides with alpha-beta unsaturated amino acid monomers. CYP121 was not observed to colocalize with other tailoring enzymes besides AlbA, and only did so in five of 176 detected clusters. The related N-methyltransferase Amir_4628 was detected in 17 clusters, among which it was found to co-occur with AlbA eight times, potentially relating to piperafazine-like products. A unique cluster featured AlbA, Amir4628, and the Ndas1145 O-/N-methyltransferase, potentially encoding an O- and N-methylated piperafazine. Ndas1145 and Ndas1149 were each identified in 15 clusters, and were found to co-occur with AlbA in all but two clusters.

Cyclodipeptide biosynthesis is incompletely understood, and relatively few tailoring reactions have been experimentally associated with CDPS clusters. To gain a broader understanding of biosynthetic transformations associated with genetically encoded cyclodipeptides, we analyzed the frequency with which other tailoring reactions within PRISM were associated with cyclodipeptide biosynthesis. PRISM contains a library of over 400 virtual tailoring reactions executed on predicted natural product scaffolds, representing a wide variety of secondary metabolic chemical transformations. All PRISM domains found within ±2.5 kb of a CDPS three or more times are plotted in Fig. 5b. The most common biosynthetic domains found in association with cyclodipeptide clusters were N-acetyltransferases and sulfotransferases, suggesting some tRNA-derived cyclodipeptides may be further modified by acetylation or sulfonation. CDPSs were also frequently found in association with thiotemplated systems, including both NRPSs and type I polyketide synthases (PKSs). Interestingly, an antibiotic monooxygenase domain associated with oxidation of aromatic polyketides was found in association with 13 CDPS clusters, all from *Amycolatopsis* spp. The absence of other type II polyketide domains from these clusters suggests this domain may catalyze a similar reaction in the context of cyclodipeptide biosynthesis. Furthermore, several models specific to enzymes involved in deoxy sugar biosynthesis, including O-methyltransferases, acetyltransferases, and epimerases, were found in clusters that also included a glycosyltransferase, primarily in *Streptomyces* isolates. This observation suggests that some clusters analyzed here may encode glycosylated cyclodipeptides.

In addition to biosynthetic domains, we also used PRISM to profile non-biosynthetic resistance domains found in close proximity to CDPSs (Fig. 5c) [21]. All six resistance domains that were found three or more times were involved in efflux, most commonly members of the major facilitator superfamily, implying a conserved mechanism for resistance to CDPS products.

Annotation of biosynthetic gene clusters within PRISM relies fundamentally on sequence similarity to experimentally characterized enzymes involved in secondary metabolite biosynthesis or resistance. We therefore used Pfam [22] to further analyze putative open reading frames (ORFs) surrounding CDPSs that were not associated with a hidden Markov model within PRISM, and manually distinguished biosynthetic and non-biosynthetic domains among the 25 most recurrent Pfam hits (Fig. 5d). Intriguingly, the two most recurrent Pfam domains within ORFs not annotated by PRISM were domains of unknown function, DUF202 and DUF2695. DUF202 is found in bacterial membrane proteins and fungal vacuolar transporter chaperones, while DUF2695 is found solely in prokaryotes. Other biosynthetic domains associated with CDPSs included FAD-linked oxidases, cytochromes P450, 2OG-Fe(II) oxygenases, and multiple families of methyltransferases, consistent with previous findings [5]. Intriguingly, among the most common non-biosynthetic Pfam domains were PAS domains, which are commonly found in signalling proteins responsible for sensing extracellular metabolites. Cyclodipeptide products encoded by these clusters may function as quorum sensing metabolites, akin to the recently discovered dipeptide signaling system in *Vibrio* [23]. Furthermore, we noted that a number of *Burkholderia* isolates possessed an operon featuring a CDPS, a single oxygenase, and several siderophore import and export pumps, indicating that the product of this gene cluster may be the first bona fide tRNA-derived cyclodipeptide siderophore. Thus, although further experimental work is required to more completely characterize the functions of these proteins, our analysis of cyclodipeptide clusters suggests common tailoring reactions, regulatory processes, and potential biological roles for their genetically encoded small molecule products.

### Functional annotation of uncharacterized ORFs associated with cyclodipeptide biosynthesis

Despite our thorough domain-based analysis of CDPS biosynthesis, a considerable number of ORFs in close proximity to CDPSs (a total of 786 within ±2.5 kb) could not be associated with either a PRISM or Pfam domain. These may represent erroneous ORF predictions, but alternatively may represent uncharacterized enzymes associated with novel biosynthetic transformations or biological processes related to the biosynthesis or biological roles of genetically encoded cyclodipeptides. We used hierarchical clustering to reveal families of unannotated ORFs, reasoning that homologous ORFs that are recurrently found in association with a CDPS are more likely to represent biologically relevant ORFs. This analysis revealed 19 clusters of ORFs found in five or more CDPS clusters, among which eight were found in ten or more (Additional file 5**:** Figure S2). We subjected these eight sets of ORFs to a further round of sequence analysis, using HHpred [24] to suggest remote homology between each multiple sequence alignment and the PDB70 database. Although the majority of these ORF clusters did not evince significant sequence similarity to any database members (as defined by an E-value less than 0.05), three alignments revealed suggestive levels of similarity to known proteins. In particular, a cluster of 19 homologous ORFs had significant sequence similarity to multiple proteins involved in cyclization of type II polyketides (E-value <10^−17^), raising the possibility that these enzymes may catalyze a similar cyclization reaction within a genetically encoded cyclodipeptide. A second cluster of 12 ORFs demonstrated similarity to leukotriene hydrolase (E-value = 1.4 × 10^−50^), and may likewise catalyze a cyclodipeptide tailoring reaction. Finally, a cluster of 14 ORFs was aligned to acetylornithine deacetylase (E-value = 8.5 × 10^−29^), an observation which may reflect incorporation of ornithine into a genetically encoded cyclodipeptide. Thus, further sequence analysis of proteins recurrently found in association with CDPSs assigns putative functions to several unannotated ORFs.

### Mapping the landscape of genetically encoded cyclodipeptides

**Fig. 6:**
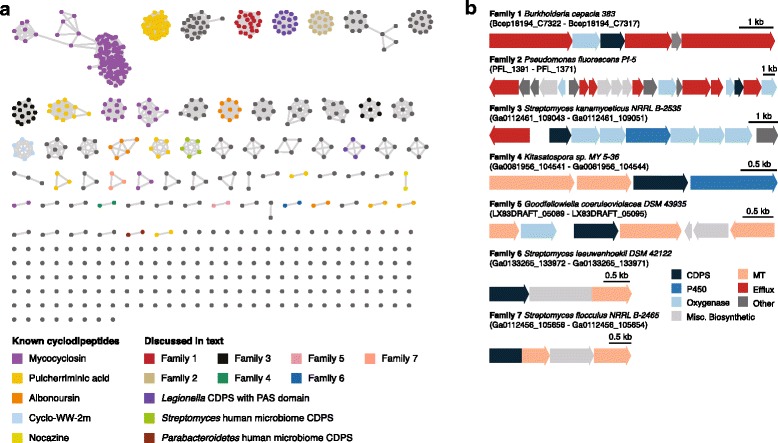
**a** Sequence similarity network of unique CDPSs encoded within 93,107 prokaryotic genomes. **b** Selected cyclodipeptide biosynthetic gene cluster families discussed in the text

Finally, we integrated genetic and chemical information to produce an integrated map of genetically encoded cyclodipeptides. We created a sequence similarity network [25] from the unique CDPS sequences, and assigned clusters to known products based on chemical structure predictions (Fig. 6). For clusters that could not be assigned to known products, we developed unique identifiers based on the conserved architecture of the cluster and the producing organisms. The 739 unique CDPSs were grouped into 68 clusters of at least two CDPSs, and 170 CDPSs that did not cluster with any other CDPS. Multiple large clusters within this network corresponded to groups of CDPSs involved in mycocyclosin or pulcherriminic acid biosynthesis. We also identified several large clusters of homologous CDPSs putatively involved in the biosynthesis of novel products. For example, two families of clusters from *Streptomyces* spp. were observed to contain terpene biosynthetic machinery and fused methyltransferases, attaching to the C-terminus of the CDPS or to a phytoene synthase, indicative of a mixed cyclodipeptide-terpenoid product. While *Kitasatospora* and *Goodfellowiella* spp. were found to possess clusters with unique combinations of methyltransferases, oxygenases, P450s, and nitroreductases, the most elaborate cluster we identified was found in *Pseudomonas*, *Tistrella*, *Streptomyces*, and several other genera, and contained 5 oxygenases along with a unique P450 enzyme.

The biological role of cyclodipeptides in their producing organisms is poorly understood. To identify relationships between cyclodipeptide biosynthesis and the human microbiome, we mapped cyclodipeptide producers to strains collected by the Human Microbiome Project (HMP) [26], revealing 31 unique CDPS clusters in HMP strains. The majority of these clusters were found in organisms that are human pathogens, including *Pseudomonas aeruginosa*, *Staphylococcus haemolyticus*, and *Porphyomonas gingivalis*. Chemical structure predictions linked the majority of nonpathogenic CDPS producers to production of pulcherriminic acid, with likely microbiome-associated pulcherriminic acid producers including *Staphylococcus lugdunensis*, *Staphylococcus epidermidis*, *Corynebacterium jeikeium*, *Bacillus sp.* 7_6_55CFAA_CT2, and *Staphylococcus hominis*. However, we also identified two CDPSs in healthy human microbiome isolates predicted to encode unknown products. The first such CDPS, produced by *Streptomyces sp.* HPH0547, was found in a cluster with a nitroreductase and methyltransferase, and clustered with five similar CDPSs from other *Streptomyces* spp. The second microbiome-associated novel CDPS was produced by *Parabacteroides sp.* 20_3, a species isolated as part of a colon biopsy of a health patient, and clustered with a related CDPS from another *Parabacteroides* species. These putatively unknown cyclodipeptides may play as-of-yet unappreciated roles in the human microbiome, and in this context their structural similarity to naturally occurring and synthetic heterocyclic molecules known to perturb human receptors or modulate signalling pathways is noteworthy [27].

## Conclusions

Among bacterial natural products, tRNA-derived cyclodipeptides are a family that has recently attracted attention for their attractive biological activities, but few user-friendly tools to facilitate genome-guided discovery of novel CDPs exist. In the present work, we have developed and validated an algorithm to identify and classify CDPS clusters, predict active site residues and aminoacyl-tRNA substrates, and finally predict the complete chemical structure of the final cluster product. Our genome-guided analysis of cyclodipeptide chemical space suggests that a considerable fraction of dipeptides encoded by these enzymes are novel, and suggests common tailoring reactions, resistance mechanisms, and potential biological roles for their products. Our pipeline is freely available through a user-friendly web application at https://magarveylab.ca/prism.

## Methods

### CDPS identification and structure prediction

We constructed a hidden Markov model composed of 273 CDPS sequences collected by Jacques et al. [14]*.* Additionally, we compiled three hidden Markov models specific to the NYH, XYP, and SYQ subfamilies, composed of 196, 76, and one sequences, respectively, based on annotations in Jacques et al. Finally, we compiled hidden Markov models for six experimentally characterized cyclodipeptide tailoring enzymes (Additional file 1**:** Table S1). Hidden Markov model compilation was performed as previously described [17]. Within PRISM, a tRNA-derived cyclodipeptide cluster is identified by the presence of a CDPS domain, with identification of clustered biosynthetic and nonbiosynthetic domains as previously described [15]. Active site residue identification is accomplished by multiple sequence alignment to all 273 CDPS sequences using MUSCLE [28], and extraction of the residues aligned to the conserved active site residues, using the AlbC CDPS as a reference. The aminoacyl-tRNA substrate of each active site is subsequently predicted using a naive Bayes classifier, as implemented within the R package e1071, using a set of 61 cyclodipeptides with known substrates compiled from Jacques et al. and independent literature review. Chemical structure prediction then proceeds from the cyclized dipeptide, with the execution of both cyclodipeptide-specific and generic tailoring reactions based on other identified biosynthetic domains within the putative cyclodipeptide cluster. The entire algorithm is integrated into the publicly available PRISM web server [18].

*Validation.* Validation of CDPS identification and classification was performed by using our previously described pipeline for hidden Markov model construction, which includes alignment, gap trimming, and HMM construction using the HMMER package, to construct new models missing the withheld sequences. Active site residue prediction was validated using the manually curated set of CDPS active sites developed by Jacques et al. [14], who used HHpred [29] to refine multiple sequence alignments based on the secondary structure of the structurally characterized AlbC enzyme. In validating the accuracy of aminoacyl-tRNA substrate prediction, we followed the example of Rausch et al. [16] by evaluating the ability of our classifier to predict the gross physicochemical properties of the substrate, in addition to its exact identity. In validating chemical structure prediction, we calculated the Tanimoto coefficient between the true product and all structures predicted by PRISM, which generates combinatorial libraries of predicted structures in the case that the site of a tailoring reaction (for example, alpha-beta unsaturation) is not unambiguously predictable. For each cluster, we calculated the median Tanimoto coefficient, and then calculated the average median Tanimoto coefficient across all known clusters. The ECFP6 fingerprint was used to calculate the Tanimoto coefficient [30].

### Global analysis of CDPS biosynthesis

PRISM version 3.1.0 was used to analyze all 93,107 prokaryotic genomes, obtained from NCBI Genome in March, 2017. The NCBI Genome database contains many identical or near-identical genomes due to repetitive sequencing, particularly of human pathogens or other human-associated strains. To eliminate bias introduced by repetitive sequencing, the resulting CDPS sequences were clustered using cd-hit [19] at an identity threshold of 100% to remove redundant sequences. This resulted in the identification of a set of unique CDPS sequences, which were retained for further analysis. We additionally explored the effect of clustering CDPS sequences at lower percent identity thresholds, in order to quantify the diversity of CDPS sequences using a strategy orthogonal to sequence similarity network analysis (Additional file 6**:** Figure S3). At a threshold of 90% similarity, only 412 unique CDPS sequences are identified. However, owing to the sensitivity of tRNA activation to the physicochemical properties of CDPS active site residues, it is no longer possible to state with certainty that these groups of CDPSs yield the same cyclodipeptide product. A single cluster (*Photorhabdus luminescens*) contained both NYH-family and XYP-family CDPSs; the NYH scored higher and so for simplicity this cluster was classified as NYH CDP in further analyses.

To identify putatively novel CDPS products within genomic sequence data, we defined the set of previously known tRNA-derived cyclodipeptides using data from Jacques et al. [14]. This list was supplemented with a set of all known cyclodipeptides by using GRAPE [20] (version 3.2.1) to perform a retrobiosynthetic analysis of all 46,165 molecules within our in-house database of known natural products [21]. Monomers within cyclic dipeptides were grouped by their general abbreviation within GRAPE (so, for example, N-methyl-serine would be grouped with serine, rather than considering it a separate monomer).

In addition to analysis with PRISM, Pfam version 31.0 was used to analyze all ORFs identified by Prodigal [31] within ±2.5 kb of a CDPS that were not also associated with a PRISM domain. Unannotated ORFs were subsequently identified as those identified by Prodigal within ±2.5 kb of a CDPS that were neither associated with PRISM or Pfam domains, and were clustered using PSI-CD-HIT [32] at an identity threshold of 10%. The resulting clusters were aligned using MUSCLE, and the alignments were used to search the PDB_mmCIF70 database using the HHpred web server.

The sequence similarity network was constructed by using BLASTP to calculate pairwise similarities between each unique CDPS. An E-value threshold of 10^−100^ was used to define the presence of an edge between any two CDPSs. The network was visualized in Cytoscape [33].

## Additional file

Additional file 1: Table S1. Hidden Markov models developed for this study, and their bit score cutoffs and associated tailoring reactions. (XLSX 41 kb)
Additional file 2: Table S2. Active site residues annotated by Jacques et al... and predicted by PRISM. (XLSX 47 kb)
Additional file 3: Figure S1. **a** Number of unique cyclodipeptide clusters found in each of 628 prokaryotic genomes. **b** Number of cyclodipeptide synthases found in each of 721 unique tRNA-derived cyclodipeptide clusters. **c** Distribution of distances between cyclodipeptide synthases, in kilobases. (PDF 347 kb)
Additional file 4: Table S3. Complete set of 739 unique cyclodipeptide synthases identified in a global analysis of publicly available prokaryotic genomes. (XLSX 144 kb)
Additional file 5: Figure S2. Distribution of unannotated open reading frame cluster sizes found in proximity to cyclodipeptide synthases. Only ORFs that clustered with at least one homologous ORF are shown. (PDF 83 kb)
Additional file 6: Figure S3. Number of unique CDPS families discovered with cd-hit as a function of percent identity threshold. (PDF 4 kb)

## Abbreviations

CDPSs: Cyclodipeptide synthases
HMP: Human Microbiome Project
Kb: Kilobases
NCBI: National Center for Biotechnology Information
NRPSs: Nonribosomal peptide synthetases
ORFs: Open reading frames
PKSs: Polyketide synthases
RiPPs: Ribosomally synthesized and post-translationally modified peptides
