# Supplementary figures and images for: Global analysis of prokaryotic tRNA-derived cyclodipeptide biosynthesis
Source: BMC Genomics. 2018 Jan 15;19:45. doi: 10.1186/s12864-018-4435-1 (PMC5767969; doi:10.1186/s12864-018-4435-1)

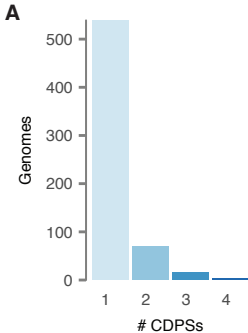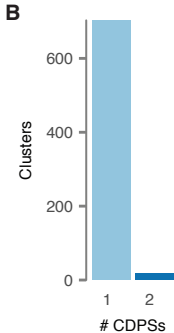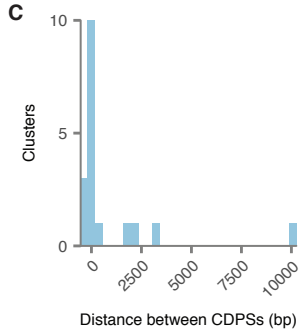

Supplement: Additional file 3: Figure S1. — a Number of unique cyclodipeptide clusters found in each of 628 prokaryotic genomes. b Number of cyclodipeptide synthases found in each of 721 unique tRNA-derived cyclodipeptide clusters. c Distribution of distances between cyclodipeptide synthases, in kilobases. (PDF 347 kb) [file 12864_2018_4435_MOESM3_ESM.pdf]

Unannotated ORF clusters

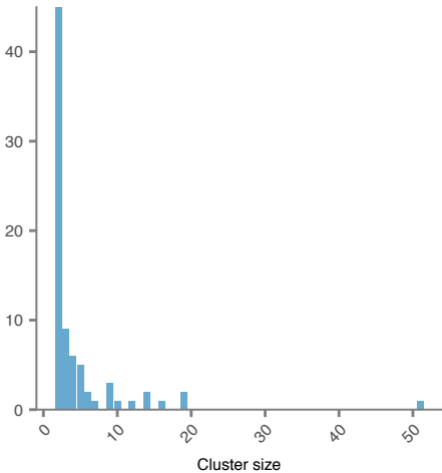

Supplement: Additional file 5: Figure S2. — Distribution of unannotated open reading frame cluster sizes found in proximity to cyclodipeptide synthases. Only ORFs that clustered with at least one homologous ORF are shown. (PDF 83 kb) [file 12864_2018_4435_MOESM5_ESM.pdf]

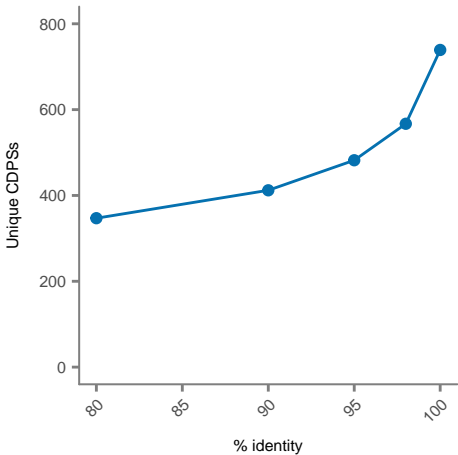

Supplement: Additional file 6: Figure S3. — Number of unique CDPS families discovered with cd-hit as a function of percent identity threshold. (PDF 4 kb) [file 12864_2018_4435_MOESM6_ESM.pdf]
